# Supplementary material for: Exploring the similarities and differences of variables collected by burn registers globally: protocol for a data dictionary review study
Source: BMJ Open. 2023 Feb 28;13(2):e066512. doi: 10.1136/bmjopen-2022-066512 (PMC9980371; doi:10.1136/bmjopen-2022-066512)
Supplement: Author's manuscript [file bmjopen-2022-066512.draft_revisions.pdf]

BMJ Open is committed to open peer review. As part of this commitment we make the peer review history of every article we publish publicly available.

When an article is published we post the peer reviewers' comments and the authors' responses online. We also post the versions of the paper that were used during peer review. These are the versions that the peer review comments apply to.

The versions of the paper that follow are the versions that were submitted during the peer review process. They are not the versions of record or the final published versions. They should not be cited or distributed as the published version of this manuscript.

BMJ Open is an open access journal and the full, final, typeset and author-corrected version of record of the manuscript is available on our site with no access controls, subscription charges or pay-per-view fees (http://bmjopen.bmj.com).

If you have any questions on BMJ Open's open peer review process please email info.bmjopen@bmj.com

BMJ Open

COMPARISON OF INTERNATIONAL BURN REGISTER DATA DICTIONARIES: PROTOCOL

Journal:	*BMJ Open*
Manuscript ID	bmjopen-2022-066512
Article Type:	Protocol
Date Submitted by the Author:	12-Jul-2022
Complete List of Authors:	Bebbington, Emily; Bangor University, Centre for Mental Health and Society; Ysbyty Gwynedd, Emergency Department<br>Miles, Joanna; Norwich University Hospitals, Plastic and Reconstructive Surgery Department<br>Peck, Michael ; Arizona Burn Center, Valleywise Health Medical Center; Creighton University Health Sciences Campus, Department of Surgery<br>Singer, Yvonne; Alfred Hospital, Victoria Adult Burn Service<br>Dunn, Ken; NHS England<br>Young, Amber; University Hospitals Bristol NHS Foundation Trust, Children's Burn Research Centre; University of Bristol, Bristol Centre for Surgical Research
Keywords:	PLASTIC & RECONSTRUCTIVE SURGERY, PUBLIC HEALTH, EPIDEMIOLOGY, International health services < HEALTH SERVICES ADMINISTRATION & MANAGEMENT, Quality in health care < HEALTH SERVICES ADMINISTRATION & MANAGEMENT

SCHOLARONE™
Manuscripts

COMPARISON OF INTERNATIONAL BURN REGISTER DATA DICTIONARIES: PROTOCOL

Emily Bebbington[1,2], Joanna Miles[3], Mike Peck[4,5], Yvonne Singer[6], Ken Dunn[7], Amber Young[8,9].

1. Centre for Mental Health and Society, Bangor University, LL57 2DG, UK.

2. Emergency Department, Ysbyty Gwynedd, Bangor, LL57 2PW, UK.

3. Plastic and Reconstructive Surgery Department, Norfolk and Norwich University Hospitals, Norwich, NR4 7UY, UK.

4. Arizona Burn Center, Valleywise Health Medical Center, Phoenix, AZ 85008, USA.

5. Department of Surgery, Creighton University Health Sciences Campus, Phoenix, AZ 85012, USA.

6. Victoria Adult Burn Service, The Alfred Hospital, Melbourne, VIC 3004, Australia.

7. Medical Director of the International Burn Injury Database, NHS England, UK.

8. Children's Burns Research Centre, The Bristol Royal Hospital for Children, Bristol, BS2 8BJ, UK.

9. Bristol Centre for Surgical Research, Population Health Sciences, Bristol Medical School, University of Bristol, Bristol, BS8 1QU, UK.

Corresponding author: Emily Bebbington, Centre for Mental Health and Society, Bangor University, LL57 2DG, UK. E.bebbington@bangor.ac.uk.

Word count: 2494 (excluding title page, abstract, references, figures and tables)

For peer review only

ABSTRACT

Introduction

Burn registers can provide high quality clinical data that can be used for surveillance, research, planning service provision, and clinical quality assessment. Pooling of international data enables investigation of rarer exposures and outcomes, tracking of emerging trends, investigation of how disease processes are affected by sociocultural factors, and embedding trials. Few international register data comparisons are completed beyond basic morbidity and mortality statistics. Data comparisons across registers require analysis of homogenous variables. Little work has been done to understand if burn registers have sufficiently comparable data to enable useful comparisons or if a common set of variables (international minimum dataset) needs to be developed.

Methods and analysis

The aim of this project is to compare the variables collected in country-wide and inter-country burn registers internationally to understand their similarities and differences. Register custodians will be invited to participate in the study and to share their register data dictionaries. Study objectives are to compare patient inclusion and exclusion criteria of each participating burn register; determine which variables are collected by each register and if variables are mandatory or optional; identify what variables are collected by all registers, and common variable themes; and compare a sample of variables to understand differences in their measurement. All variable names will be extracted from each register and common themes identified. Detailed information will be extracted for a sample of variables to give a deeper insight into similarities and differences between registers.

Ethics and dissemination

No patient data will be used in this project. Permission to use each register’s data dictionary will be sought from respective register custodians. Results will be presented at international meetings and published in open access journals. These results will be of interest to register custodians and researchers wishing to explore international data comparisons, and countries wishing to establish their own burn register.

For peer review only

STRENGTHS AND LIMITATIONS OF THIS STUDY

Strengths

- Training and pilot exercises will be undertaken to ensure data extraction is completed to a high standard.
- Extracted data will be verified by a second researcher.
- A custodian from each register will be invited to be part of the study team to ensure accurate interpretation of the data dictionaries, subsequent analyses, and write up.

Limitations

- Registers were identified from the peer reviewed literature. Registers may have been missed if they have not published their findings.
- The majority of burn registers exist in high income countries. This study may underrepresent variables important for burn prevention and care in lower resourced environments.

For peer review only

INTRODUCTION

Approximately 9 million people globally sustain burn injuries each year requiring medical treatment, of whom 120 000 die.[1] Over 80% of these injuries and deaths occur in low and middle-income countries.[1] Lower-income countries often have poor coverage of surveillance data, meaning that the true burden of disease in these countries is not fully known.[1,2] Where data is collected, it frequently does not include information required to inform prevention and intervention strategies, such as disaggregation of data by injury intent in regions where there are high rates of deliberate burns.[1,3-6] Burn mortality surveillance statistics are compiled from civil registration and vital statistic data, whereas burn morbidity statistics are calculated from hospital based data.[2]

Burn registers provide clinical data that can be used for international morbidity surveillance. However, few international burn data comparisons are completed beyond basic morbidity and mortality statistics. Registers can be used for outcomes assessment, research, planning service provision, clinical governance, quality improvement, service accreditation or, as clinical quality registers, to identify variation in practice.[7] The utility of burn registers is such that there are now numerous country-wide registers (e.g. Dutch Burn Repository) as well as inter-country registers (e.g. Burn registry of Australia and New Zealand collects data from Australia and New Zealand, International Burn Injury Database collects data from England and Wales, German Burn Registry collects data from German speaking countries, and National Burn Repository collects data from US centres and some international burn centres).[8-12] Established burn registers are strongly concentrated in high income countries, most likely due to ethical, regulatory, technological, and economic issues.[13] A notable exception is the World Health Organisation Global Burn Registry (WHO GBR).[14] This register allows any healthcare facility globally to submit and analyse their data for free. Twenty countries submit data to the WHO GBR, the majority of which are middle-income countries.[15] Most countries that submit data do not have a country-wide burn register. The success of the WHO GBR in countries without an active burn register likely reflects the enthusiasm of the international burn community for rigorously collected and collated burn injury data.

The variables collected by burn registers are not standardised internationally, thereby limiting international data comparisons. The development of a set of variables that are collected across all registers in a standardised way (an international minimum dataset) would allow the comparison of data on issues of international significance. Pooling data from registers effectively achieves a larger sample size allowing investigation of rarer exposures and outcomes, tracking of emerging trends, investigation of how disease processes are affected by sociocultural factors, and

For peer review only

embedding trials.[16-21] Custodians of a country-wide or inter-country register might choose to incorporate an international minimum dataset into existing data collection processes to help facilitate international data comparisons, but is likely to continue to collect country specific variables required to tailor prevention strategies, service provision, and quality improvement to the long-term needs of the population covered.

It is not known whether burn registers already collect any variables in a way that would allow international data comparisons. If data is not comparable, an international minimum dataset would need to be developed as differences between registers may represent sources of bias during analyses. To achieve this will require internationally agreed variable definitions and methods of measurement. It is necessary to understand which variables are commonly collected across all registers prior to the development of an international minimum dataset as it is likely that important common themes at present may not be collected in comparable ways. Agreed definitions would also be helpful to countries wishing to establish their own burn registers. Little work has been done to understand the similarities and differences across burn registers internationally. The aim of this project is to compare the variables collected in country-wide and inter-country burn registers internationally to understand their similarities and differences.

METHODS AND ANALYSIS

The study objectives are to:

1. Compare patient inclusion and exclusion criteria of each participating burn register
2. Determine what variables are collected by each register and if variables are mandatory or optional
3. Identify if any variables are collected by all registers and identify common variable themes
4. Undertake a detailed comparison of a sample of variables to understand differences in definitions and measurement methods.

The steps of the study are shown in Figure 1. No reporting guidelines for protocols or studies of this nature were found on the Equator Network website (www.equator-network.org). Any deviations from the study protocol will be reported in the results manuscript.

Terminology

For peer review only

Common terms applicable to the study have been defined to ensure uniformity of understanding across international collaborators (Table 1). Standard definitions have been used where possible. These definitions will be used in all research material and manuscripts.

Table 1. Definitions of key terms used in this study.

Term	Definition	Example
Registry	An organisation and associated systems that support the upkeep of a register.[7]	World Health Organisation Global Burn Registry
Register custodian	Organisation responsible for maintaining reliability and security of a register's data.[22]	World Health Organisation
Register	A physical or electronic collection of pre-specified and systematically recorded details.[7]	Details about burn presentations to a hospital
Variable	One feature of interest in a register.	'Date of birth', 'total body surface area of burn' (TBSA), 'discharge disposition'.
Variable response options	Potential choices to answer a variable.	Response options for 'discharge disposition' may include 'discharged home', 'transferred', 'discharged against medical advice', 'died'.
Mandatory variable	A variable that must be inputted by the person completing data entry.	These are specific to each register and are dependent on the analyses that are deemed essential. Essential variables might include 'date of birth', 'TBSA'.
Optional variable	A variable that is not collected about every patient.	These are specific to each register and are dependent on the analyses that are deemed important but not essential. For example 'income', 'occupation'.
Minimum data set	The list of mandatory variables collected by the register	Specific to each register.
Electronic database	Collection of data organised for rapid search and retrieval by a computer.[23]	SQL
Calculated field	A piece of information that is computed using variable data.	Age may be calculated using the variable 'date of birth'.
Data dictionary	Document that defines each variable, their limits, and validation parameters.[24-26]	Specific to each register. May use standard definitions for a variable such as International Classification of Diseases 11th Revision.

For peer review only

Outcome	Variable measured at a specific time point to assess the efficacy or harm of an intervention.[27]	Quality of life
Outcome measure	Method of quantifying an outcome of interest.	EQ-5D instrument

Eligibility criteria for burn register participation in the study

Country-wide and inter-country burn registers will be identified from a scoping review of active burn registers.[28,29] A register will be classed as country-wide or inter-country if there is the potential for healthcare facilities across a single country or multiple countries to submit data. Registers that are restricted to a single state or region of a country will be excluded in countries with an active country-wide or inter-country register. Burn register pilot studies will be included for countries that do not have an active burn register to ensure wider representation of low and middle-income countries. Contact information from the register website will be used to invite register custodians to participate in the study. In cases where there is no register website, corresponding authors of recent register publications will be contacted to provide up to date information about the register custodian. Each custodian will be asked to provide a copy of their most recent data dictionary or equivalent document that explains which variables are collected by the burn register. Registers that have freely available data dictionaries will be automatically included in the study.

Data handling and storage

No patient identifiable information will be collected. Data dictionaries and associated research data will be held on an encrypted cloud storage system to allow international collaboration across institutional boundaries. Access to the cloud storage system will be agreed by the authors and permissions set accordingly.

Data extraction rationale and process

Objective 1. Compare patient inclusion and exclusion criteria of each participating burn register.

Each register uses a set of inclusion and exclusion criteria to determine which patients will have their information recorded in the burn register. For example, a register may include only patients with a burn injury requiring admission for more than 24 hours and exclude patients receiving care on an outpatient basis. Inclusion and exclusion criteria will be compared between registers to understand the patient populations under study. Inclusion criteria

For peer review only

heterogeneity could represent a source of selection bias if data were compared without allowing for this. One author (EB) will extract patient inclusion and exclusion criteria from each register's data dictionary into a spreadsheet file. Where this information is not included in the data dictionary, it will be sought from the register website or custodian (order of preference). 100% of the data will be verified by a second author (JM).

Objectives 2 and 3. Determine what variables are collected by each register and if variables are mandatory or optional. Identify if any variables are collected by all registers and identify common variable themes.

A diverse range of mandatory and optional information is collected about patients in burn registers. We will collate and compare mandatory and optionally collected variables noting differences between them. Calculated fields will not be included because they are a form of analysis reflecting the expertise of the data analyst rather than the raw data collected by the register. All variable names from the data dictionaries will be extracted by a researcher (JM) and 100% verified by a second researcher (EB). Data will be extracted into a spreadsheet file. The same extracted data will be used for objective 2 and 3.

Objective 4. Undertake a detailed comparison of a sample of variables to understand differences in definitions and measurement methods.

Data about the same topic may be collected by each register in different ways. For example, information on the intent of the burn injury may include multiple variables such as patient reported injury intent, physician suspicion of injury intent, and International Classification of Diseases external causes of morbidity codes. Each register may use different definitions for the variable and include different response options. Comparison of data that has been collected using different definitions, methods, or timing of measurement would represent a potential source of misclassification bias.

Detailed information will be extracted for a sample of topics. These will include 'patient age', 'timing of injury', 'injury cause', 'injury intent', 'infection', and 'survival'. The topics are chosen because they are likely to be collected by all registers. All variables related to each topic will be extracted. This will allow more comprehensive comparisons of variables across registers.

A pilot exercise will be completed in two phases to ensure the detailed variable information is extracted accurately. Firstly, two researchers (EB/JM) will extract detailed information on a sample of 20 variables from two freely

For peer review only - http://bmjopen.bmj.com/site/about/guidelines.xhtml

available data dictionaries. This will allow the development and refinement of the data extraction form – a spreadsheet file with predefined column headings. Codes will be developed to ensure that a reason is assigned for missing data in the data extraction form. Secondly, data extraction for the topics 'patient age' and 'timing of injury' will be completed independently by two researchers (EB/JM) into the data extraction form. Interrater reliability will be calculated. Providing a good level of agreement is reached (Kappa statistic >0.60), data extraction will then be split equally between the two researchers (EB – injury cause and injury intent, JM – infection and survival). Regular discussion will be held to ensure any method developments are documented and applied universally. These will be reported in the final paper.

Data analysis, synthesis, and presentation

A custodian from each register will be invited to be part of the study team to ensure accurate interpretation of the data dictionaries, analyses, and write up.

Objective 1. Compare patient inclusion and exclusion criteria of each participating burn register

A table will be presented in the main manuscript that includes the register name, countries contributing to the register, and patient inclusion and exclusion criteria.

Objective 2. Determine what variables are collected by each register and if variables are mandatory or optional

A full list of variables will be presented for each register with mandatory and optional variables reported differently. Summary data on the number of variables (mandatory and optional) collected by each register will be presented in tabular format in the main manuscript to understand the differences in volume of data collection occurring internationally.

Objective 3. Identify if any variables are collected by all registers and identify common variable themes

Variables will be divided into iteratively developed clinically meaningful thematic groups (e.g. injury causation and severity) and subgroups (e.g. injury intent, size of burn) by two researchers (EB/JM) and checked by a third (AY). Variables in each group will be compared to identify if any are collected by all registers in the same way. These will be listed in the manuscript. Common themes across registers will be presented as a figure (e.g. word cloud or Venn diagram).

For peer review only

Objective 4. Undertake a detailed comparison of a sample of variables to understand differences in definitions and measurement methods.

Detailed information collected by each register for variables relating to the topics 'patient age', 'timing of injury', 'injury cause', 'injury intent', 'infection', and 'survival' will be compared across registers and presented in tables. Comparisons will be made about the variable definitions, response options, timing of measurement, and method of measurement.

PATIENT AND PUBLIC INVOLVEMENT

A member of the public with experience of burns service planning and commissioning has reviewed this manuscript for readability and to ensure the needs of the service user are represented.

ETHICS AND DISSEMINATION

No patient data will be used in this project therefore ethical approval is not required. Permissions will be requested from each register custodian to analyse data dictionaries where these dictionaries are not freely available. Prospective registration has not been completed as there is no appropriate register for this study type.

Results will be presented at international academic meetings to reach interested stakeholder groups (e.g. International Society for Burn Injuries World Congress). Peer reviewed publications of results will be published open access where possible to ensure accessibility. Custodians will be encouraged to disseminate the results in their country or territory. The collaboration formed for this study will be the basis for working together to address any recommendations for future work from the results manuscript.

AUTHOR CONTRIBUTIONS

All authors devised the study. EB/JM/KD/AY refined the methods. EB wrote the first draft of the manuscript. All authors reviewed the manuscript and agreed on the final version.

FUNDING

This article presents independent research funded by the UK National Institute for Health Research (NIHR) Advanced Research Fellowship NIHR 301362. The views expressed are those of the authors and not necessarily those of the United Kingdom National Health Service, the NIHR or the United Kingdom Department of Health. The study was also

For peer review only - http://bmjopen.bmj.com/site/about/guidelines.xhtml

supported by the NIHR Biomedical Research Centre at the University Hospitals Bristol and Weston NHS Foundation Trust and the University of Bristol.

COMPETING INTERESTS

EB, JM, MP, and YS do not declare any conflicts of interest. KD is the medical director of the International Burn Injury Database (iBID). As part of this role he is the co-chair of the Burn Care Informatics Group. This is a part time role funded by NHS England. AY is funded to undertake a priority setting exercise and a registry based trial with the iBID registry. This work is part of work funded by the National Institute of Health Research Advanced Fellowship awarded to Dr Amber Young (Ref NIHR301362). The views expressed are those of the author(s) and not necessarily those of the NIHR or the Department of Health and Social Care. AY has received funding for a cohort study on the impact of genetic make-up on scarring after small area scalds from the Scar Free Foundation. AY is a minority stakeholder in a small-medium sized enterprise on burn wound infection. She has not received any money or expenses from this.

ACKNOWLEDGEMENTS

The authors thank Mr Roy Dudley-Southern MBE for their time reviewing the manuscript and their useful comments.

WORD COUNT

2494 excluding title page, abstract, tables, and references.

REFERENCES

1. James SL, Lucchesi LR, Bisignano C, et al. Epidemiology of injuries from fire, heat and hot substances: global, regional and national morbidity and mortality estimates from the Global Burden of Disease 2017 study. *Injury Prevention* 2020;26(Supp 1):i36. doi: 10.1136/injuryprev-2019-043299
2. Vos T, Lim SS, Abbafati C, et al. Global burden of 369 diseases and injuries in 204 countries and territories, 1990–2019: a systematic analysis for the Global Burden of Disease Study 2019. *The Lancet* 2020;396(10258):1204-22. doi: 10.1016/S0140-6736(20)30925-9
3. Laloe V. Patterns of deliberate self-burning in various parts of the world. A review. *Burns* 2004;30(3):207-15.
4. Rezaeian M. The geographical belt of self-immolation. *Burns* 2017;43(5):896-97. doi: https://doi.org/10.1016/j.burns.2017.01.001
5. Bhalla K, Sanghavi P. Fire-related deaths among women in India are underestimated. *The Lancet* 2020;395(10226):779-80. doi: 10.1016/S0140-6736(20)30060-X
6. Sanghavi P, Bhalla K, Das V. Fire-related deaths in India in 2001: a retrospective analysis of data. *Lancet* 2009;373(9671):1282-8. doi: https://dx.doi.org/10.1016/S0140-6736(09)60235-X
7. Weddell J. Registers and Registries: A Review. *International Journal of Epidemiology* 1973;2(3):221-28. doi: 10.1093/ije/2.3.221
8. Taylor SL, Lee D, Nagler T, et al. A Validity Review of the National Burn Repository. *Journal of Burn Care & Research* 2013;34(2):274-80. doi: 10.1097/BCR.0b013e3182642b46
9. iBID. int. burn Injury Database. Available from: https://www.ibidb.org/ibid-introduction/January 2022.
10. Monash University. Burns Registry of Australia and New Zealand (BRANZ). Available from: https://www.monash.edu/medicine/sphpm/branzJanuary 2022.

For peer review only - http://bmjopen.bmj.com/site/about/guidelines.xhtml

11. Thamm OC, Lefering R, Bialke M, et al. Establishment of the German Burn Registry - five years of prospective data collection. *Burns* 2022 doi: https://doi.org/10.1016/j.burns.2022.02.010
12. Kahn SA, Bernal N, Mosier MJ. Pearls From the National Burn Repository. *Journal of Burn Care & Research* 2018;39(4):626-27. doi: 10.1093/jbcr/irx049
13. Wyber R, Vaillancourt S, Perry W, et al. Big data in global health: improving health in low- and middle-income countries. *Bull World Health Organ* 2015;93(3):203-08. doi: 10.2471/BLT.14.139022
14. Peck M, Falk H, Meddings D, et al. The design and evaluation of a system for improved surveillance and prevention programmes in resource-limited settings using a hospital-based burn injury questionnaire. *Inj Prev* 2016;22 Suppl 1(Suppl 1):i56-62. doi: https://doi.org/10.1136/injuryprev-2015-041815
15. World Health Organisation. Global Burn Registry. Available from: https://www.who.int/teams/social-determinants-of-health/safety-and-mobility/burns/global-burn-registry accessed 08/05/2022.
16. Avillach P, Coloma PM, Gini R, et al. Harmonization process for the identification of medical events in eight European healthcare databases: the experience from the EU-ADR project. *Journal of the American Medical Informatics Association* 2013;20(1):184-92. doi: 10.1136/amiajnl-2012-000933
17. Hripcsak G, Duke JD, Shah NH, et al. Observational Health Data Sciences and Informatics (OHDSI): Opportunities for Observational Researchers. *Stud Health Technol Inform* 2015;216:574-78. https://pubmed.ncbi.nlm.nih.gov/26262116
18. Voss EA, Makadia R, Matcho A, et al. Feasibility and utility of applications of the common data model to multiple, disparate observational health databases. *Journal of the American Medical Informatics Association* 2015;22(3):553-64. doi: 10.1093/jamia/ocu023
19. Chazard E, Merlin B, Ficheur G, et al. Detection of adverse drug events: proposal of a data model. *Stud Health Technol Inform* 2009;148:63-74.
20. Weeks J, Pardee R. Learning to Share Health Care Data: A Brief Timeline of Influential Common Data Models and Distributed Health Data Networks in U.S. Health Care Research. *EGEMS (Wash DC)* 2019;7(1):4. doi: 10.5334/egems.279
21. Danese MD, Halperin M, Duryea J, et al. The Generalized Data Model for clinical research. *BMC Medical Informatics and Decision Making* 2019;19(1):117. doi: 10.1186/s12911-019-0837-5
22. GOV.UK. Registers: becoming a custodian GOV.UK. Available from: https://www.gov.uk/guidance/registers-becoming-a-custodianJanuary 2022.
23. Encyclopaedia Britannica. Database: Britannia. January 2022.
24. Stow PJ, Hart GK, Higlett T, et al. Development and implementation of a high-quality clinical database: the Australian and New Zealand Intensive Care Society Adult Patient Database. *Journal of Critical Care* 2006;21(2):133-41. doi: https://doi.org/10.1016/j.jcrc.2005.11.010
25. Arts DGT, de Keizer NF, Scheffer G-J. Defining and Improving Data Quality in Medical Registries: A Literature Review, Case Study, and Generic Framework. *Journal of the American Medical Informatics Association* 2002;9(6):600-11. doi: 10.1197/jamia.M1087
26. Linnarsson R, Wigertz O. The data dictionary--a controlled vocabulary for integrating clinical databases and medical knowledge bases. *Methods Inf Med* 1989;28(2):78-85.
27. Chan A-W, Krleza-Jerić K, Schmid I, et al. Outcome reporting bias in randomized trials funded by the Canadian Institutes of Health Research. *CMAJ* 2004;171(7):735-40. doi: 10.1503/cmaj.1041086
28. Brooks S. Burn registry state of affairs: a scoping review: Open Science Framework, 2021.
29. Gus E, Brooks S, Multani I, et al. Burn Registries State of Affairs: A Scoping review. *Journal of Burn Care & Research* 2022 doi: 10.1093/jbcr/irac077

For peer review only

FIGURE LEGEND

Figure 1. The process of register recruitment, data extraction, and data analyses that the study will follow.

For peer review only

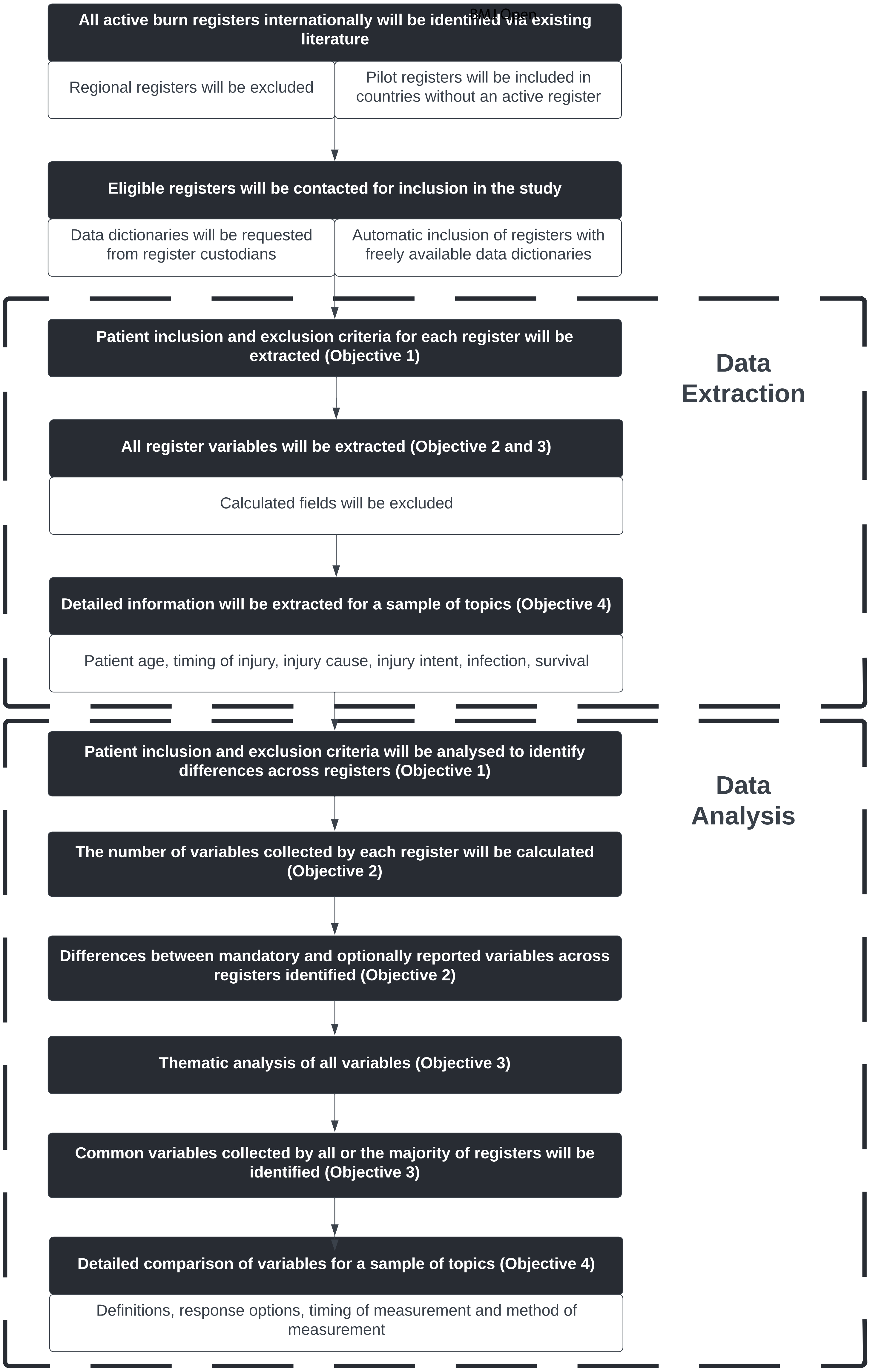
All active burn registers internationally will be identified via existing literature
Regional registers will be excluded
Pilot registers will be included in countries without an active register
Eligible registers will be contacted for inclusion in the study
Data dictionaries will be requested from register custodians
Automatic inclusion of registers with freely available data dictionaries
Data Extraction
Patient inclusion and exclusion criteria for each register will be extracted (Objective 1)
All register variables will be extracted (Objective 2 and 3)
Calculated fields will be excluded
Detailed information will be extracted for a sample of topics (Objective 4)
Patient age, timing of injury, injury cause, injury intent, infection, survival
Data Analysis
Patient inclusion and exclusion criteria will be analysed to identify differences across registers (Objective 1)
The number of variables collected by each register will be calculated (Objective 2)
Differences between mandatory and optionally reported variables across registers identified (Objective 2)
Thematic analysis of all variables (Objective 3)
Common variables collected by all or the majority of registers will be identified (Objective 3)
Detailed comparison of variables for a sample of topics (Objective 4)
Definitions, response options, timing of measurement and method of measurement

BMJ Open

EXPLORING THE SIMILARITIES AND DIFFERENCES OF VARIABLES COLLECTED BY BURN REGISTERS GLOBALLY: PROTOCOL FOR A DATA DICTIONARY REVIEW STUDY

Journal:	*BMJ Open*
Manuscript ID	bmjopen-2022-066512.R1
Article Type:	Protocol
Date Submitted by the Author:	23-Jan-2023
Complete List of Authors:	Bebbington, Emily; Bangor University, Centre for Mental Health and Society; Ysbyty Gwynedd, Emergency Department<br>Miles, Joanna; Norwich University Hospitals, Plastic and Reconstructive Surgery Department<br>Peck, Michael ; Arizona Burn Center, Valleywise Health Medical Center; Creighton University Health Sciences Campus, Department of Surgery<br>Singer, Yvonne; Alfred Hospital, Victoria Adult Burn Service<br>Dunn, Ken; NHS England<br>Young, Amber; University Hospitals Bristol NHS Foundation Trust, Children's Burn Research Centre; University of Bristol, Bristol Centre for Surgical Research
**Primary Subject Heading**:	Surgery
Secondary Subject Heading:	Epidemiology, Global health, Public health
Keywords:	PLASTIC & RECONSTRUCTIVE SURGERY, PUBLIC HEALTH, EPIDEMIOLOGY, International health services < HEALTH SERVICES ADMINISTRATION & MANAGEMENT, Quality in health care < HEALTH SERVICES ADMINISTRATION & MANAGEMENT

SCHOLARONE™
Manuscripts

EXPLORING THE SIMILARITIES AND DIFFERENCES OF VARIABLES COLLECTED BY BURN REGISTERS GLOBALLY: PROTOCOL FOR A DATA DICTIONARY REVIEW STUDY

Emily Bebbington[1,2], Joanna Miles[3], Michael Peck[4,5], Yvonne Singer[6], Ken Dunn[7], Amber Young[8,9].

1. Centre for Mental Health and Society, Bangor University, LL57 2DG, UK.

2. Emergency Department, Ysbyty Gwynedd, Bangor, LL57 2PW, UK.

3. Plastic and Reconstructive Surgery Department, Norfolk and Norwich University Hospitals, Norwich, NR4 7UY, UK.

4. Arizona Burn Center, Valleywise Health Medical Center, Phoenix, AZ 85008, USA.

5. Department of Surgery, Creighton University Health Sciences Campus, Phoenix, AZ 85012, USA.

6. Victoria Adult Burn Service, The Alfred Hospital, Melbourne, VIC 3004, Australia.

7. Medical Director of the International Burn Injury Database, NHS England, UK.

8. Children's Burns Research Centre, The Bristol Royal Hospital for Children, Bristol, BS2 8BJ, UK.

9. Bristol Centre for Surgical Research, Population Health Sciences, Bristol Medical School, University of Bristol, Bristol, BS8 1QU, UK.

Corresponding author: Emily Bebbington, Centre for Mental Health and Society, Bangor University, LL57 2DG, UK. E.bebbington@bangor.ac.uk.

Word count: 2563 (excluding title page, abstract, references, figures and tables)

For peer review only

ABSTRACT

Introduction

Burn registers can provide high quality clinical data that can be used for surveillance, research, planning service provision, and clinical quality assessment. Many country-wide and inter-country burn registers now exist. The variables collected by burn registers are not standardised internationally. Few international burn register data comparisons are completed beyond basic morbidity and mortality statistics. Data comparisons across registers require analysis of homogenous variables. Little work has been done to understand if burn registers have sufficiently similar variables to enable useful comparisons. The aim of this project is to compare the variables collected in country-wide and inter-country burn registers internationally to understand their similarities and differences.

Methods and analysis

Register custodians will be invited to participate in the study and to share their register data dictionaries. Study objectives are to compare patient inclusion and exclusion criteria of each participating burn register; determine which variables are collected by each register and if variables are required or optional, identify common variable themes; and compare a sample of data variables to understand how they are defined and measured. All variable names will be extracted from each register and common themes identified. Detailed information will be extracted for a sample of variables to give a deeper insight into similarities and differences between registers.

Ethics and dissemination

No patient data will be used in this project. Permission to use each register's data dictionary will be sought from respective register custodians. Results will be presented at international meetings and published in open access journals. These results will be of interest to register custodians and researchers wishing to explore international data comparisons, and countries wishing to establish their own burn register.

For peer review only

STRENGTHS AND LIMITATIONS OF THIS STUDY

- Training and pilot exercises will be undertaken to ensure register design data extraction is completed to a high standard.
- Extracted information will be verified by a second researcher.
- A custodian from each register will be invited to be part of the study team to ensure accurate interpretation of the data dictionaries, subsequent analyses, and write up.
- Registers will be identified from the peer reviewed literature, which may miss those that have not published their findings.
- The majority of burn registers exist in high income countries, so this study may underrepresent variables important for burn prevention and care in lower resourced environments.

For peer review only

INTRODUCTION

Approximately 9 million people globally sustain burn injuries each year requiring medical treatment, of whom 120 000 die.[1] Over 80% of these injuries and deaths occur in low and middle-income countries.[1] Lower-income countries often have poor coverage of surveillance data, meaning that the true burden of disease in these countries is not fully known.[1,2] Where data is collected, it frequently does not include information required to inform prevention and intervention strategies, such as disaggregation of data by injury intent in regions where there are high rates of deliberate burns.[1,3-6] Burn mortality surveillance statistics are compiled from civil registration and vital statistic data, whereas burn morbidity statistics are calculated from hospital based data.[2]

Burn registers provide clinical data that can be used for international morbidity surveillance. However, few international burn data comparisons are completed beyond basic morbidity and mortality statistics. Registers can be used for outcomes assessment, research, planning service provision, clinical governance, quality improvement, service accreditation or, as clinical quality registers, to identify variation in practice.[7] The utility of burn registers is such that there are now numerous country-wide registers (e.g. Dutch Burn Repository) as well as inter-country registers (e.g. Burn registry of Australia and New Zealand collects data from Australia and New Zealand, International Burn Injury Database collects data from England and Wales, German Burn Registry collects data from German speaking countries, and National Burn Repository collects data from US centres and some international burn centres).[8-12] Established burn registers are strongly concentrated in high income countries, most likely due to ethical, regulatory, technological, and economic issues.[13] A notable exception is the World Health Organisation Global Burn Registry (WHO GBR).[14] This register allows any healthcare facility globally to submit and analyse their data for free. Twenty countries submit data to the WHO GBR, the majority of which are middle-income countries.[15] Most countries that submit data do not have a country-wide burn register. The success of the WHO GBR in countries without an active burn register likely reflects the enthusiasm of the international burn community for rigorously collected and collated burn injury data.

The variables collected by burn registers are not standardised internationally, thereby limiting international data comparisons. The development of a set of variables that are collected across all registers in a standardised way (an international minimum dataset) would allow the comparison of data on issues of international significance. Pooling data from registers effectively achieves a larger sample size allowing investigation of rarer exposures and outcomes, tracking of emerging trends, investigation of how disease processes are affected by sociocultural factors, and embedding trials.[16-21] Custodians of a country-wide or inter-country register might choose to incorporate an

For peer review only

international minimum dataset into existing data collection processes to help facilitate international data comparisons, but are likely to continue to collect country specific variables required to tailor prevention strategies, service provision, and quality improvement to the long-term needs of their population.

It is not known whether burn registers already collect any variables in a way that would allow international data comparisons. If data is not comparable, an international minimum dataset would need to be developed as differences between registers may represent sources of bias during analyses. To achieve this will require internationally agreed variable definitions and methods of measurement. It is necessary to understand which variables are commonly collected across all registers prior to the development of an international minimum dataset as it is likely that important common themes at present may not be collected in comparable ways. Agreed definitions would also be helpful to countries wishing to establish their own burn registers. Little work has been done to understand the similarities and differences across burn registers internationally. The aim of this project is to compare the variables collected in country-wide and inter-country burn registers internationally to understand their similarities and differences.

METHODS AND ANALYSIS

The study objectives are to:

1. Compare patient inclusion and exclusion criteria of each participating burn register
2. Determine what variables are collected by each register and if variables are required or optional
3. Identify if any variables are collected by all registers and identify common variable themes
4. Undertake a detailed comparison of a sample of variables to understand differences in definitions and measurement methods.

The steps of the study are shown in Figure 1. No reporting guidelines for protocols or studies of this nature were found on the Equator Network website (www.equator-network.org). Any deviations from the study protocol will be reported in the results manuscript.

Terminology

Common terms applicable to the study have been defined to ensure uniformity of understanding across international collaborators (Table 1). Standard definitions have been used where possible. These definitions will be used in all research material and manuscripts.

For peer review only

Table 1. Definitions of key terms used in this study.

Term	Definition	Example
Registry	An organisation and associated systems that support the upkeep of a register.[7]	World Health Organisation Global Burn Registry
Register custodian	Organisation responsible for maintaining reliability and security of a register's data.[22]	World Health Organisation
Register	A physical or electronic collection of pre-specified and systematically recorded details.[7]	Details about burn presentations to a hospital
Data dictionary	Document that defines each variable in a register, their limits, and validation parameters.[23-25] It does not include any patient data.	Specific to each register. May use standard definitions for a variable such as International Classification of Diseases 11th Revision.
Variable	One feature of interest in a register.	'Date of birth', 'total body surface area of burn' (TBSA), 'discharge disposition'.
Variable response options	Potential choices to answer a variable.	Response options for 'discharge disposition' may include 'discharged home', 'transferred', 'discharged against medical advice', 'died'.
Required variable	A variable that must be inputted by the person completing data entry.	These are specific to each register and are dependent on the analyses that are deemed essential. Essential variables might include 'date of birth', 'TBSA'.
Optional variable	A variable that is not collected about every patient.	These are specific to each register and are dependent on the analyses that are deemed important but not essential. For example 'income', 'occupation'.
Minimum data set	The list of required variables collected by the register	Specific to each register.
Electronic database	Collection of data organised for rapid search and retrieval by a computer.[26]	SQL
Calculated field	A piece of information that is computed using variable data.	Age may be calculated using the variable 'date of birth'.
Outcome	Variable measured at a specific time point to assess the efficacy or harm of an intervention.[27]	Quality of life
Outcome measure	Method of quantifying an outcome of interest.	EQ-5D instrument

For peer review only

Eligibility criteria for burn register participation in the study

Country-wide and inter-country burn registers will be invited to participate in the study. Registers will be identified from a scoping review of active burn registers.[28,29] A register will be classed as country-wide or inter-country if there is the potential for healthcare facilities across a single country or multiple countries to submit data. The WHO GBR meets these inclusion criteria despite data submission being locally rather than nationally coordinated. The variables included in the WHO GBR are of particular importance because of the wide uptake of the WHO GBR in low- and middle-income countries, which are underrepresented in burn register studies. Registers that are restricted to a single state or region of a country will be excluded in countries with an active country-wide or inter-country register. Burn register pilot studies will be included for countries that do not have an active burn register to attempt to further increase representation of low and middle-income countries. Contact information from the register website will be used to invite register custodians to participate in the study. In cases where there is no register website, corresponding authors of recent register publications will be contacted to provide up to date information about the register custodian. Each custodian will be asked to provide a copy of their most recent data dictionary or equivalent document that explains which variables are collected by the burn register. Registers that have freely available data dictionaries will be automatically included in the study.

Data handling and storage

No patient data will be collected. Data dictionaries and project documents will be held on an encrypted cloud storage system to allow international collaboration across institutional boundaries. Access to the cloud storage system will be agreed by the authors and permissions set accordingly.

Register design data extraction rationale and process

Objective 1. Compare patient inclusion and exclusion criteria of each participating burn register.

Each register uses a set of inclusion and exclusion criteria to determine which patients will have their information recorded in the burn register. For example, a register may include only patients with a burn injury requiring admission for more than 24 hours and exclude patients receiving care on an outpatient basis. Inclusion and exclusion criteria will be compared between registers to understand the patient populations under study. Inclusion criteria heterogeneity could represent a source of selection bias if data were compared without allowing for this. One author

For peer review only - http://bmjopen.bmj.com/site/about/guidelines.xhtml

(EB) will extract patient inclusion and exclusion criteria from each register's data dictionary into a spreadsheet file. Where this information is not included in the data dictionary, it will be sought from the register website or custodian (order of preference). 100% of the data will be verified by a second author (JM).

Objectives 2 and 3. Determine what variables are collected by each register and if variables are required or optional. Identify if any variables are collected by all registers and identify common variable themes.

A diverse range of required and optional information is collected about patients in burn registers. We will collate and compare required and optionally collected variables noting differences between them. Calculated fields will not be included because they are a form of analysis reflecting the expertise of the data analyst rather than the raw data collected by the register. All variable names from the data dictionaries will be extracted by a researcher (JM) and 100% verified by a second researcher (EB). Data will be extracted into a spreadsheet file. The same extracted data will be used for objective 2 and 3.

Objective 4. Undertake a detailed comparison of a sample of variables to understand differences in definitions and measurement methods.

Data about the same topic may be collected by each register in different ways. For example, information on the intent of the burn injury may include multiple variables such as patient reported injury intent, physician suspicion of injury intent, and International Classification of Diseases external causes of morbidity codes. Each register may use different definitions for the variable and include different response options. Comparison of data that has been collected using different definitions, methods, or timing of measurement would represent a potential source of misclassification bias.

Detailed information will be extracted from the data dictionaries for a sample of topics. These will include 'patient age', 'timing of injury', 'injury cause', 'injury intent', 'infection', and 'survival'. The topics are chosen because they are likely to be collected by all registers. All variables related to each topic will be extracted. This will allow more comprehensive comparisons of variables across registers.

A pilot exercise will be completed in two phases to ensure the detailed variable information is extracted accurately. Firstly, two researchers (EB/JM) will extract detailed information on a sample of 20 variables from two freely available data dictionaries. This will allow the development and refinement of the data dictionary extraction form – a spreadsheet file with predefined column headings (e.g. variable name, variable definition, method of measurement).

For peer review only

Codes will be developed to ensure that a reason is assigned for missing fields in the extraction form. Secondly, detailed variable extraction for the topics 'patient age' and 'timing of injury' will be completed independently by two researchers (EB/JM) using the extraction form. Interrater reliability will be calculated. Providing a good level of agreement is reached (Kappa statistic >0.60), data dictionary extraction will then be split equally between the two researchers (EB – injury cause and injury intent, JM – infection and survival). Regular discussion will be held to ensure any method developments are documented and applied universally. These will be reported in the final paper.

Analysis, synthesis, and presentation

A custodian from each register will be invited to be part of the study team to ensure accurate interpretation of the data dictionaries, analyses, and write up.

Objective 1. Compare patient inclusion and exclusion criteria of each participating burn register

A table will be presented in the main manuscript that includes the register name, countries contributing to the register, and patient inclusion and exclusion criteria.

Objective 2. Determine what variables are collected by each register and if variables are required or optional

A full list of variables will be presented for each register with required and optional variables reported differently. Summary data on the number of variables (required and optional) collected by each register will be presented in tabular format in the main manuscript to understand the differences in volume of data collection occurring internationally.

Objective 3. Identify if any variables are collected by all registers and identify common variable themes

Variables will be divided into iteratively developed clinically meaningful thematic groups (e.g. injury causation and severity) and subgroups (e.g. injury intent, size of burn) by two researchers (EB/JM) and checked by a third (YS). Variables in each group and subgroup will be compared to identify the most commonly collected variable themes across registers and if any are variables are collected by all registers in the same way. These will be listed in the manuscript. Common themes across registers will be presented as a figure.

Objective 4. Undertake a detailed comparison of a sample of variables to understand differences in definitions and measurement methods.

For peer review only

Detailed information collected by each register for variables relating to the topics ‘patient age’, ‘timing of injury’, ‘injury cause’, ‘injury intent’, ‘infection’, and ‘survival’ will be compared across registers and presented in tables. Comparisons will be made about the variable definitions, response options, timing of measurement, and method of measurement.

PATIENT AND PUBLIC INVOLVEMENT

A member of the public with experience of burns service planning and commissioning has reviewed this manuscript for readability and to ensure the needs of the service user are represented.

ETHICS AND DISSEMINATION

This project involves the analysis of burn register data dictionaries and freely available burn register related documents (e.g. website, research papers) only. No human participant data will be used in this project, therefore ethical approval is not required in accordance with UK Research and Innovation, and the Declaration of Helsinki.[30,31] Permissions will be requested from each register custodian to analyse data dictionaries where these dictionaries are not freely available. Only data dictionaries that we have express permission to use will be included in the project. Data will be anonymised and aggregated as required. The identity of the register will only be given with permission of the custodian. Prospective registration has not been completed as there is no appropriate register for this study type.

Results will be presented at international academic meetings to reach interested stakeholder groups (e.g. International Society for Burn Injuries World Congress). Peer reviewed publications of results will be published open access where possible to ensure accessibility. Custodians will be encouraged to disseminate the results in their country or territory. The collaboration formed for this study will be the basis for working together to address any recommendations for future work from the results manuscript.

AUTHOR CONTRIBUTIONS

EB, JM, MP, YS, KD, and AY conceived the study and refined the study methods that are presented in this protocol manuscript. EB wrote the first draft of the manuscript. All authors reviewed the manuscript and agreed on the final version. EB and KD act as guarantors of the work.

FUNDING

For peer review only

This article presents independent research funded by the UK National Institute for Health Research (NIHR) Advanced Research Fellowship NIHR 301362. The views expressed are those of the authors and not necessarily those of the United Kingdom National Health Service, the NIHR or the United Kingdom Department of Health. The study was also supported by the NIHR Biomedical Research Centre at the University Hospitals Bristol and Weston NHS Foundation Trust and the University of Bristol.

COMPETING INTERESTS

EB, JM, MP, and YS do not declare any conflicts of interest. KD is the medical director of the International Burn Injury Database (iBID). As part of this role he is the co-chair of the Burn Care Informatics Group. This is a part time role funded by NHS England. AY is funded to undertake a priority setting exercise and a registry based trial with the iBID registry. This work is part of work funded by the National Institute of Health Research Advanced Fellowship awarded to Dr Amber Young (Ref NIHR301362). The views expressed are those of the author(s) and not necessarily those of the NIHR or the Department of Health and Social Care. AY has received funding for a cohort study on the impact of genetic make-up on scarring after small area scalds from the Scar Free Foundation. AY is a minority stakeholder in a small-medium sized enterprise on burn wound infection. She has not received any money or expenses from this.

ACKNOWLEDGEMENTS

Sadly, in the time between submission and publication of this paper Professor Amber Young passed away. The co-authors would like to acknowledge her tremendous contribution to this project and the burn community in general. The authors also thank Mr Roy Dudley-Southern MBE for his time reviewing the manuscript and his useful comments.

WORD COUNT

2563 excluding title page, abstract, tables, and references.

REFERENCES

1. James SL, Lucchesi LR, Bisignano C, et al. Epidemiology of injuries from fire, heat and hot substances: global, regional and national morbidity and mortality estimates from the Global Burden of Disease 2017 study. *Injury Prevention* 2020;26(Supp 1):i36. doi: 10.1136/injuryprev-2019-043299
2. Vos T, Lim SS, Abbafati C, et al. Global burden of 369 diseases and injuries in 204 countries and territories, 1990–2019: a systematic analysis for the Global Burden of Disease Study 2019. *The Lancet* 2020;396(10258):1204-22. doi: 10.1016/S0140-6736(20)30925-9
3. Laloe V. Patterns of deliberate self-burning in various parts of the world. A review. *Burns* 2004;30(3):207-15. doi: https://doi.org/10.1016/j.burns.2003.10.018

For peer review only

4. Rezaeian M. The geographical belt of self-immolation. *Burns* 2017;43(5):896-97. doi: https://doi.org/10.1016/j.burns.2017.01.001
5. Bhalla K, Sanghavi P. Fire-related deaths among women in India are underestimated. *The Lancet* 2020;395(10226):779-80. doi: 10.1016/S0140-6736(20)30060-X
6. Sanghavi P, Bhalla K, Das V. Fire-related deaths in India in 2001: a retrospective analysis of data. *Lancet* 2009;373(9671):1282-8. doi: https://doi.org/10.1016/s0140-6736(09)60235-x
7. Weddell J. Registers and Registries: A Review. *International Journal of Epidemiology* 1973;2(3):221-28. doi: 10.1093/ije/2.3.221
8. Taylor SL, Lee D, Nagler T, et al. A Validity Review of the National Burn Repository. *Journal of Burn Care & Research* 2013;34(2):274-80. doi: 10.1097/BCR.0b013e3182642b46
9. iBID. int. burn Injury Database. Available from: https://www.ibidb.org/ibid-introduction/January 2022.
10. Monash University. Burns Registry of Australia and New Zealand (BRANZ). Available from: https://www.monash.edu/medicine/sphpm/branzJanuary 2022.
11. Thamm OC, Lefering R, Bialke M, et al. Establishment of the German Burn Registry - five years of prospective data collection. *Burns* 2022 doi: https://doi.org/10.1016/j.burns.2022.02.010
12. Kahn SA, Bernal N, Mosier MJ. Pearls From the National Burn Repository. *Journal of Burn Care & Research* 2018;39(4):626-27. doi: 10.1093/jbcr/irx049
13. Wyber R, Vaillancourt S, Perry W, et al. Big data in global health: improving health in low- and middle-income countries. *Bull World Health Organ* 2015;93(3):203-08. doi: 10.2471/BLT.14.139022
14. Peck M, Falk H, Meddings D, et al. The design and evaluation of a system for improved surveillance and prevention programmes in resource-limited settings using a hospital-based burn injury questionnaire. *Inj Prev* 2016;22 Suppl 1(Suppl 1):i56-62. doi: https://doi.org/10.1136/injuryprev-2015-041815
15. World Health Organisation. Global Burn Registry. Available from: https://www.who.int/teams/social-determinants-of-health/safety-and-mobility/burns/global-burn-registry accessed 08/05/2022.
16. Avillach P, Coloma PM, Gini R, et al. Harmonization process for the identification of medical events in eight European healthcare databases: the experience from the EU-ADR project. *Journal of the American Medical Informatics Association* 2013;20(1):184-92. doi: 10.1136/amiajnl-2012-000933
17. Hripcsak G, Duke JD, Shah NH, et al. Observational Health Data Sciences and Informatics (OHDSI): Opportunities for Observational Researchers. *Stud Health Technol Inform* 2015;216:574-78. https://pubmed.ncbi.nlm.nih.gov/26262116
18. Voss EA, Makadia R, Matcho A, et al. Feasibility and utility of applications of the common data model to multiple, disparate observational health databases. *Journal of the American Medical Informatics Association* 2015;22(3):553-64. doi: 10.1093/jamia/ocu023
19. Chazard E, Merlin B, Ficheur G, et al. Detection of adverse drug events: proposal of a data model. *Stud Health Technol Inform* 2009;148:63-74.
20. Weeks J, Pardee R. Learning to Share Health Care Data: A Brief Timeline of Influential Common Data Models and Distributed Health Data Networks in U.S. Health Care Research. *EGEMS (Wash DC)* 2019;7(1):4. doi: 10.5334/egems.279
21. Danese MD, Halperin M, Duryea J, et al. The Generalized Data Model for clinical research. *BMC Medical Informatics and Decision Making* 2019;19(1):117. doi: 10.1186/s12911-019-0837-5
22. GOV.UK. Registers: becoming a custodian GOV.UK. Available from: https://www.gov.uk/guidance/registers-becoming-a-custodianJanuary 2022.
23. Stow PJ, Hart GK, Higlett T, et al. Development and implementation of a high-quality clinical database: the Australian and New Zealand Intensive Care Society Adult Patient Database. *Journal of Critical Care* 2006;21(2):133-41. doi: https://doi.org/10.1016/j.jcrc.2005.11.010
24. Arts DGT, de Keizer NF, Scheffer G-J. Defining and Improving Data Quality in Medical Registries: A Literature Review, Case Study, and Generic Framework. *Journal of the American Medical Informatics Association* 2002;9(6):600-11. doi: 10.1197/jamia.M1087
25. Linnarsson R, Wigertz O. The data dictionary--a controlled vocabulary for integrating clinical databases and medical knowledge bases. *Methods Inf Med* 1989;28(2):78-85.
26. Encyclopaedia Britannica. Database: Britannia. January 2022.
27. Chan A-W, Krleza-Jerić K, Schmid I, et al. Outcome reporting bias in randomized trials funded by the Canadian Institutes of Health Research. *CMAJ* 2004;171(7):735-40. doi: 10.1503/cmaj.1041086
28. Brooks S. Burn registry state of affairs: a scoping review: Open Science Framework, 2021.
29. Gus E, Brooks S, Multani I, et al. Burn Registries State of Affairs: A Scoping review. *Journal of Burn Care & Research* 2022 doi: 10.1093/jbcr/irac077

30. UKRI Medical Research Council. Understanding health research: Ethical approval. Available from: https://www.ukri.org/councils/mrc/facilities-and-resources/find-an-mrc-facility-or-resource/mrc-regulatory-support-centre/understanding-health-research/ethical-approval/ accessed January 2023.
31. World Medical Association. WMA DECLARATION OF HELSINKI – ETHICAL PRINCIPLES FOR MEDICAL RESEARCH INVOLVING HUMAN SUBJECTS. In: World Medical Association, ed., 2013.

For peer review only

FIGURE LEGEND

Figure 1. The process of register recruitment, data extraction, and data analyses that the study will follow.

For peer review only

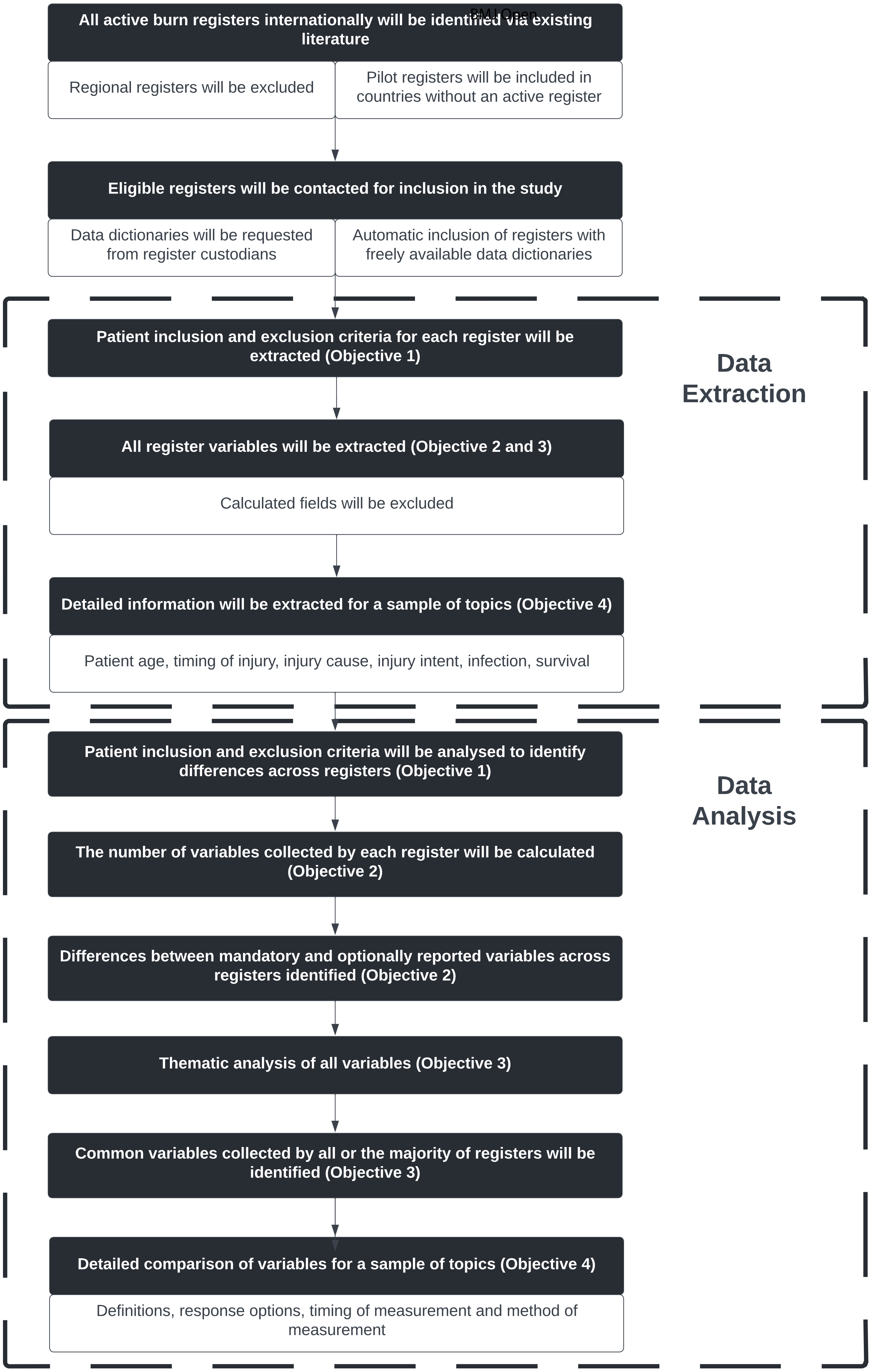
All active burn registers internationally will be identified via existing literature
Regional registers will be excluded
Pilot registers will be included in countries without an active register
Eligible registers will be contacted for inclusion in the study
Data dictionaries will be requested from register custodians
Automatic inclusion of registers with freely available data dictionaries
Data Extraction
Patient inclusion and exclusion criteria for each register will be extracted (Objective 1)
All register variables will be extracted (Objective 2 and 3)
Calculated fields will be excluded
Detailed information will be extracted for a sample of topics (Objective 4)
Patient age, timing of injury, injury cause, injury intent, infection, survival
Data Analysis
Patient inclusion and exclusion criteria will be analysed to identify differences across registers (Objective 1)
The number of variables collected by each register will be calculated (Objective 2)
Differences between mandatory and optionally reported variables across registers identified (Objective 2)
Thematic analysis of all variables (Objective 3)
Common variables collected by all or the majority of registers will be identified (Objective 3)
Detailed comparison of variables for a sample of topics (Objective 4)
Definitions, response options, timing of measurement and method of measurement
